# Pure malignant rhabdoid tumor of the left kidney in an adult: A case report and review of the literature

**DOI:** 10.3892/ol.2013.1207

**Published:** 2013-02-22

**Authors:** GUANGNING ZHAO, REN NA, YUMING YANG, RUIFA HAN

**Affiliations:** 1 Department of Urology, The Second Hospital of Tianjin Medical University, Tianjin Institute of Urology, Tianjin 300211;; 2 Department of Clinical Laboratory, Tianjin Children’s Hospital, Tianjin 300074, P.R. China

**Keywords:** renal, malignant, rhabdoid tumor, adult

## Abstract

Malignant rhabdoid tumors of the kidney (MRTKs) are extremely rare. Pure MRTKs in adult patients are particularly rare and have not been previously reported in China. Due to the non-specific clinical symptoms, it is difficult but also essential to be able to give a definite diagnosis. The present study reports a case of pure adult malignant rhabdoid tumor in a patient’s left kidney with characteristic clinicopathological features. Considering the fact that the characteristic findings are often not observed in clinical symptom and imaging studies, the histological features, immunohistochemical staining and cytogenetic studies may aid in confirming the diagnosis of pure MRTKs. Although pure adult MRTKs remain extremely uncommon, it is necessary to consider this possibility when such types of renal tumors are encountered.

## Introduction

Malignant rhabdoid tumors of the kidney (MRTKs) are uncommon renal tumors which mainly occur in children and are extremely rare in adult patients. The present study describes a malignant rhabdoid tumor identified in a patient’s left kidney and the diagnosis which was made mainly based on characteristic histological findings and immunohistochemical features. To the authors’ knowledge, only five adult cases have been reported in the English-language literature and the present case is the first report of adult MRTK in China (1–5). We propose that this study is likely play a significant role in guiding clinical practice in the diagnosis and therapy of MRTKs.

The study was approved by the ethics committee of the Second Hospital of Tianjin Medical University, Tianjin Institute of Urology (Tianjin, China). Informed consent was obtained from the patient prior to the study.

## Case report

**Figure 1:**
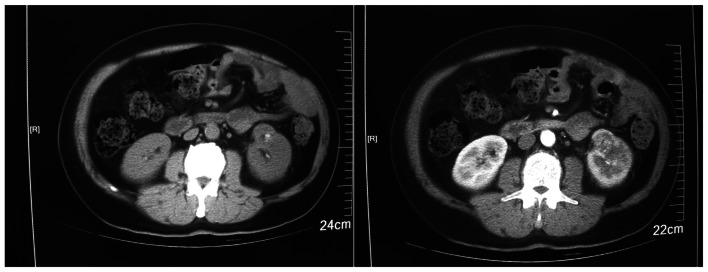
Abdominal computed tomography scan. A computed tomography scan revealed a renal mass with a mixed density in the lower pole of the left kidney measuring 5×4×5 cm.

A 59-year-old male patient was admitted to the Second Hospital of Tianjin Medical University due to an asymptomatic mass located in the left kidney which was revealed by ultrasonography one week before. The patient had no other symptoms with the exception of a weight loss of 6 kg over the past months. Physical and laboratory examination revealed no abnormalities. Ultrasonography revealed the presence of a mass, measuring 4.9×3.8×5.0 cm in the left kidney, with medium echo, as well as an unclear boundary and internal inhomogeneous echo. A computed tomography scan revealed a mixed density renal mass in the lower pole of the left kidney, measuring 5×4×5 cm. The lesion exhibited clear peripheral enhancement at the cortical phase and a low density at the parenchymal and delayed phase. There was also increased density in the left perirenal fat. In addition, the following were observed: a shadow with liquid density in the left pleural cavity, nodules with ring-enhancement posterior to the left costophrenic angle and lymph node enlargement around the left abdominal aorta (Fig. 1). A chest radiograph revealed a group of flakes with increased density in the left lung field.

**Figure 2:**
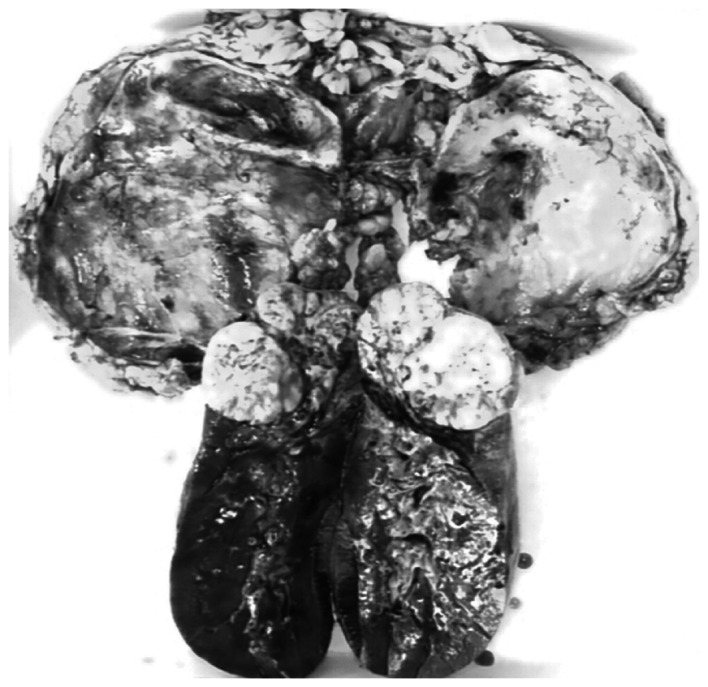
Macroscopic features of MRTK. When cut, the firm mass was white to grayish with a fleshy texture and focal areas of necrosis and hemorrhage. MRTK, malignant rhabdoid tumor of the kidney.

An initial diagnosis of a space-occupying lesion of the left kidney, retroperitoneal and left costophrenic angle lymph node metastases and a space-occupying lesion of the left lung was proposed (with suspicions of renal cell carcinoma; the clinical TNM staging was T3aN2M1) and subsequently, the patient underwent left radical nephrectomy. Surgery revealed one mass in the lower pole of the kidney which extended locally into the capsule. The cut surface of the tumor was white to gray with a fleshy texture and focal areas of necrosis and hemorrhage (Fig. 2).

**Figure 3:**
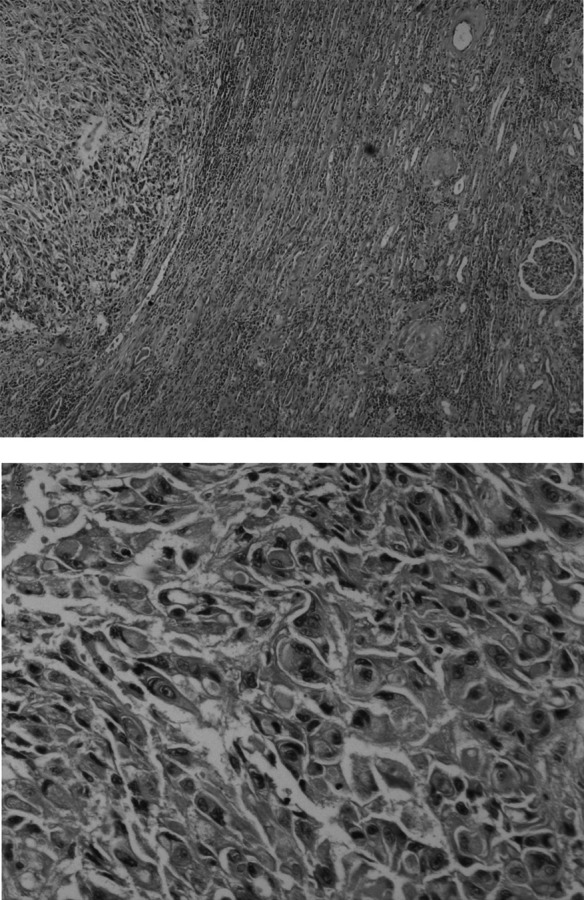
Histological features of MRTK. The tumor cells exhibited large, ovoid or round to polygonal shapes in a noncohesive pattern with eosinophilic cytoplasm; the tumor cells had vesicular nuclei and prominent nucleoli; certain neoplastic cells had eccentric nuclei and large round eosinophilic cytoplasmic inclusion-like structures (hematoxylin and eosin staining). MRTK, malignant rhabdoid tumor of the kidney.

**Figure 4:**
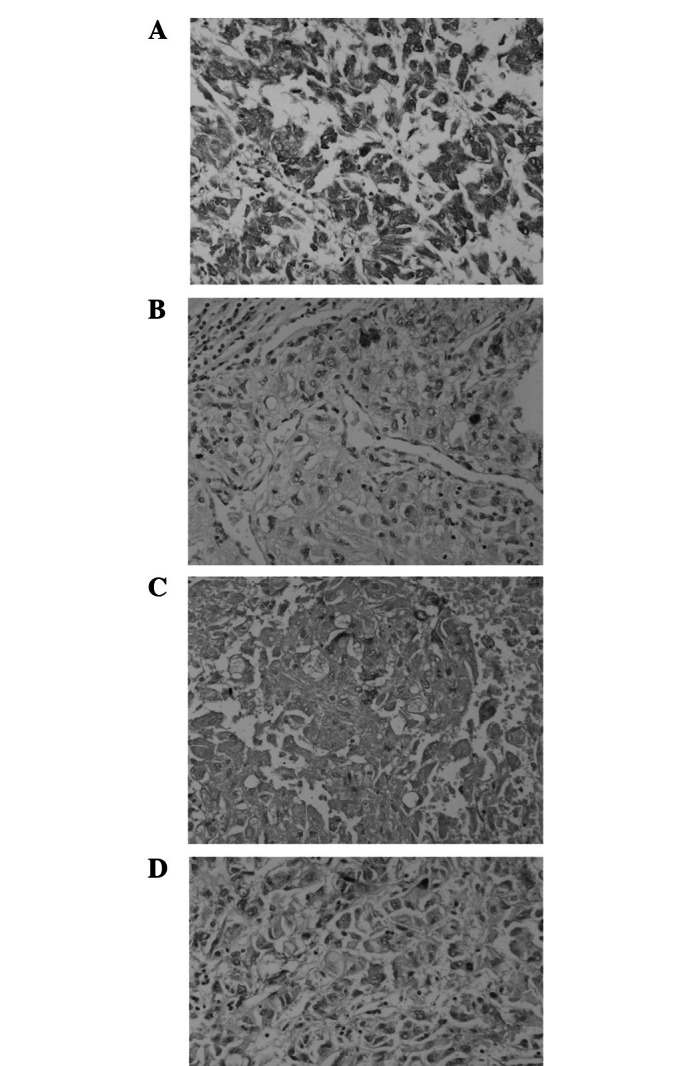
Immunohistochemical features of MRTK. The tumor cells were (A) diffusely positive for vimentin and focally positive (cytoplasmic and dot-like) for (B) NSE, (C) S-100 and (D) EMA (immunohistochemical staining). MRTK, malignant rhabdoid tumor of the kidney; NSE, neuron-specific enolase; EMA, epithelial membrane antigen.

Microscopically, the tumor cells were noncohesive, large and round to polygonal, with vesicular nuclei and prominent nucleoli. A number of the neoplastic cells had eccentric nuclei and large round eosinophilic cytoplasmic inclusion-like structures. Certain areas of the tumor exhibited hemorrhage and/or necrosis (Fig. 3A and B). Immunohistochemically, the tumor cells were diffusely positive for vimentin (Fig. 4A) and focally positive (cytoplasmic and dotlike) for neuron-specific enolase (NSE; Fig. 4B), S-100 (Fig. 4C) and epithelial membrane antigen (EMA; Fig. 4D). The staining was negative for pan-cytokeratin, CK7, myoglobin, desmin, muscle-specific actin (MSA) and smooth muscle actin (SMA).

Accordingly, a pathological diagnosis of pure malignant rhabdoid tumor of the left kidney was established based on the microscopic features and immunohistochemical findings.

A 10-month postoperative follow-up revealed no indications of tumor recurrence or metastasis.

## Discussion

MRTKs were originally described as a ‘rhabdomyosarcomatoid variant of Wilm’s tumor’ by Beckwith and Palmer in 1978 due to the resemblance of the cells to rhabdomyoblasts (6). Subsequently, this type of tumor was recognized as a distinct and unique malignant renal tumor. MRTK has also been described at extrarenal sites in children and adults, including the urogenital system such as the bladder or prostate (7–9).

MRTK may be associated with other malignancies (composite) or constitute the only component (pure). Several carcinomas arising in the kidney have been observed to have rhabdoid features, including renal clear cell, papillary, chromophobe, transitional cell and collecting duct carcinomas (10–12). However, only five cases of pure adult MRTK have been reported in the English-language literature. The present study describes the sixth case of pure adult MRTK.

Patients typically exhibit an abdominal mass but only a minority have hematuria and flank or abdominal pain. In a small subset of patients the tumor is discovered incidentally in radiological examination (1–5). However, haematuria is a common symptom in children. Symptoms arising from metastases are common at diagnosis since metastases occur in ∼80% of patients, mainly affecting the lungs, liver and brain. Patients may also experience reversible hypercalcaemia as a result of increased parathyroid hormone concentration (13). The characteristic findings of imaging studies are often not present in adult patients, whereas CT findings suggesting rhabdold tumors of kidney, including calcification, subcapsular hematoma and the lobular appearance of a large, centrally located heterogeneous renal mass, are often observed in children (14). In the present case, the patient did not have the perceived clinical symptoms and no characteristics were observed in the imaging studies. Thus, the asymptomatic mass may have been be at an early stage.

Macroscopically, MRTK is generally a single, poorly circumscribed mass, located in the middle of the kidney which exhibits infiltrating growth. Sections of MRTKs are white to grayish with fleshy or soft textures. Necrosis and hemorrhage foci may also be observed. The classic histological appearance of MRTK is that of patternless sheets of noncohesive, large, ovoid or round to polygonal cells with vesicular nuclei and prominent nucleoli, with certain neoplastic cells exhibiting eccentric nuclei and large round eosinophilic cytoplasmic inclusion-like structures. These were all consistent with the present case. In addition, certain inflammatory cells, particularly lymphocytes or histiocytes, may accompany the neoplastic cells and the rhabdoid elements are of high nuclear grade. Ultrastructurally, the rhabdoid cells have paranuclear intermediate filament aggregates, which are arranged haphazardly. In addition, paranuclear condensation of organelles associated with peripheral vacuolization also occurs (1–5,15,16).

Immunohistochemically, the tumor cells are frequently stained diffusely positive for vimentin and focally positive for EMA, cytokeratin, NSE and S-100. However, staining for myoglobin, desmin, MSA, SMA, human melanoma, black-45 (HMB-45) and CD99 are often negative (1–5,15,16), which is consistent with the present case.

Molecular studies have revealed an apparently characteristic presence of mutations in the hSNF5/INI1 gene on chromosome 22, which has become the hallmark of cranial, renal and other rhabdoid tumors, particularly in children. The lack of immunohistochemical staining for the INI1 gene product facilitates the diagnosis of such diseases. In adults, the rhabdoid phenotype is viewed as a non-specific morphological feature due to the ‘dedifferentiation’ common to numerous neoplasms, including various carcinomas, sarcomas and meningiomas. These tumors do not exhibit the deletion of the h5NF5/INI1 gene so the nuclear immunostaining for INI1 is retained. However, it remains possible to identify the inactivation of h5NF5/INI1 in certain adult MRTs when properly tested. Consequently, whether all tumors within one individual exhibit the downregulation of hSNF5/INI1 remains controversial (17).

In the present case, although electron microscopic examination and genetic and/or molecular analysis were not performed due to technical limitations at the time, a pathological diagnosis of pure MRTK was established based on the histological and immunohistochemical analysis, whereas several previous researchers have based the diagnosis of rhabdoid tumors solely on morphological and immunohistochemical findings (1–3).

Surgery is considered to be the first choice of treatment if possible. The former postoperative treatment for MRTK cases in children was radiotherapy or a four-drug regimen for high risk nephroblastoma patients (18). However, considering the dismal outcome of full chemotherapy or radiotherapy, more effective treatments such as molecular-targeted or neoadjuvant therapy approaches are highly desirable. Kapoor *et al*’s study reported a response to sorafenib with overall disease stabilization in a patient with adult rhabdoid RCC, indicating that tyrosine kinase inhibitors may be used for effective therapeutic strategies (19). Koos *et al* noted that cell lines obtained from rhabdoid tumors of the kidney and extrarenal rhabdoid tumors consistently expressed the tyrosine kinase c-Abl. Treatment with the tyrosine kinase inhibitor imatinib, resulted in reduced cellular growth in the two cell lines. This study demonstrated a functional role for c-Abl in the biology of rhabdoid tumors and provided a rationale for the investigation of tyrosine kinase inhibitors that target c-Abl for the treatment of these tumors (20). Venneti *et al* observed that MRTs expressed numerous stem cell-associated transcription factors, which may be regulated by the expression of EZH2 and the Id family of proteins. This study demonstrated similarities between MRTs and stem cells and it may aid in the elucidation of common biological pathways that could serve in advancing more effective therapeutic strategies for treating MRTs (21). These studies all indicate that a deeper understanding of the biology of these aggressive tumors is likely to aid in the design of more effective therapies.

The prognosis is generally poor for MRTKs and the majority recur and/or metastasize. Studies indicate that there are no differences in survival due to the primary site, gender, or ethnicity in children. The stage of the tumor is negatively correlated with survival, while the use of radiotherapy is positively associated with survival. In addition, patients younger than 2 or older than 18 years at diagnosis have lower survival rates than patients between 2 and 18 years. Adult patients have improved outcomes compared with young children (<2 years old) but a poorer outcome compared with older children (2–18 years old). The tumor stage significantly affects the outcome but the use of radiotherapy is not associated with the difference in outcome (17). In the present case, tumor recurrence or metastasis were not observed at the 10-month postoperative follow-up. However, long term follow-up is essential since metastases may occur after several years in view of the malignancy.

In conclusion, the present study reports one case of pure adult MRTK with characteristic clinicopathological features. Considering the fact that the characteristic findings are often not observed in clinical symptom and imaging studies, the histological features, immunohistochemical staining and cytogenetic studies may aid in confirming the diagnosis of pure MRTK. In addition, further study and a larger case series are required to fully clarify the natural history and identify the ideal postoperative treatment protocol for these aggressive tumors.

## References

[1] Lowe W, Weiss RM, Todd MB, True LD (1990). Malignant rhabdoid tumor of the kidney in an adult. J Urol.

[2] Clausen HV, Horn T, Anagnostaki L, Larsen S (1994). Malignant rhabdoid tumour of the kidney in an adult: a case report with immunohistochemical and ultrastructural investigation. Scand J Urol Nephrol Suppl.

[3] Ebbinghaus SW, Herrera G, Marshall ME (1995). Rhabdoid tumor of the kidney in an adult: review of the literature and report of a case responding to interleukin-2. Cancer Biother.

[4] Caballero JM, Collera P, Marti L (1996). Malignant rhabdoid tumor of the kidney in the adult. Actas Urol Esp.

[5] Peng HQ, Stanek AE, Teichberg S (2003). Malignant rhabdoid tumor of the kidney in an adult: a case report and review of the literature. Arch Pathol Lab Med.

[6] Beckwith JB, Palmer NF (1978). Histopathology and prognosis of Wilms’ tumor results from the first National Wilms’ Tumor Study. Cancer.

[7] Palmer NF, Sutow W (1983). Clinical aspects of the rhabdoid tumor of the kidney: a report of the National Wilms’ Tumor Study Group. Med Pediatr Oncol.

[8] Savage N, Linn D, McDonough C (2012). Molecularly confirmed primary malignant rhabdoid tumor of the urinary bladder: implications of accurate diagnosis. Ann Diagn Pathol.

[9] Kim JY, Cho YM, Ro JY (2010). Prostatic stromal sarcoma with rhabdoid features. Ann Diagn Pathol.

[10] Kumar S, Kumar D, Cowan DF (1992). Transitional cell carcinoma with rhabdoid features. Am J Surg Pathol.

[11] Weeks DA, Beckwith JB, Mierau GW, Zuppan CW (1991). Renal neoplasms mimicking rhabdoid tumor of kidney. A report from the National Wilms’ Tumor Study Pathology Center. Am J Surg Pathol.

[12] Yusuf Y, Belmonte AH, Tchertkoff V (1996). Fine needle aspiration cytology of a recurrent malignant tumor of the kidney with rhabdoid fetures in an adult. A case report. Acta Cytol.

[13] Ahmed HU, Arya M, Levitt G (2007). Part I: Primary malignant non-Wilms’ renal tumours in children. Lancet Oncol.

[14] Chung CJ, Lorenzo R, Rayder S (1995). Rhabdoid tumor of the kidney in children: CT findings. AJR Am J Roentgenol.

[15] Gökden N, Nappi O, Swanson PE (2000). Renal cell carcinoma with rhabdoid features. Am J Surg Pathol.

[16] Shannon B, Stan Wisniewski Z, Bentel J, Cohen RJ (2002). Adult rhabdoid renal cell carcinoma. Arch Pathol Lab Med.

[17] Sultan I, Qaddoumi I, Rodríguez-Galindo C (2010). Age, stage, and radiotherapy, but not primary tumor site, affects the outcome of patients with malignant rhabdoid tumors. Pediatr Blood Cancer.

[18] van den Heuvel-Eibrink MM, van Tinteren H, Rehorst H (2011). Malignant rhabdoid tumours of the kidney (MRTKs), registered on recent SIOP protocols from 1993 to 2005: a report of the SIOP renal tumour study group. Pediatr Blood Cancer.

[19] Kapoor A, Tutino R, Kanaroglou A, Hotte SJ (2008). Treatment of adult rhabdoid renal cell carcinoma with sorafenib. Can Urol Assoc J.

[20] Koos B, Jeibmann A, Lünenbürger H (2010). The tyrosine kinase c-Abl promotes proliferation and is expressed in atypical teratoid and malignant rhabdoid tumors. Cancer.

[21] Venneti S, Le P, Martinez D (2011). Malignant rhabdoid tumors express stem cell factors, which relate to the expression of EZH2 and Id proteins. Am J Surg Pathol.

